# A Comparative Study of Food Intake and Adipose Tissue Distribution in Saudi Women with Polycystic Ovarian Syndrome

**DOI:** 10.3390/healthcare12030369

**Published:** 2024-02-01

**Authors:** Nujud H. Hurayb, Ghedeir M. Alshammari, Abdulrahman S. Al-Khalifa, Nora Alafif, Dania H. Aljaroudi, Mohammed A. Mohammed, Abu ElGasim Ahmed Yagoub, Mohammed Abdo Yahya

**Affiliations:** 1 Department of Food Science and Nutrition, College of Food and Agricultural Sciences, King Saud University, Riyadh 11451, Saudi Arabia; njooud95hj@gmail.com (N.H.H.); akhalifa@ksu.edu.sa (A.S.A.-K.); 442106434@student.ksu.edu.sa (M.A.M.); amohammed4@ksu.edu.sa (A.E.A.Y.); mabdo@ksu.edu.sa (M.A.Y.); 2 Department of Community Health Sciences, College of Applied Medical Sciences, King Saud University, Riyadh 11534, Saudi Arabia; nalafeef@ksu.edu.sa; 3 Research Center King Fahad Medical City (KFMC), P.O. Box 59046, Riyadh 11525, Saudi Arabia; daljroudi@kfmc.med.sa

**Keywords:** polycystic ovarian syndrome, sociodemographic, food intake, anthropometric indices, lipid profile

## Abstract

Polycystic ovary syndrome (PCOS) is a frequent disorder that affects reproductive-aged women and has reproductive, metabolic, and psychosocial effects. This research was intended to investigate the comparison between food intake and adipose tissue distribution in Saudi women suffering from PCOS and a control group. To determine the sociodemographic variables, a case–control study was performed with patients from King Fahad Medical City’s Reproductive Endocrine and Infertility Medicine Department (REIMD). The case–control study comprised 42 PCOS patients (PCOS-Ps) and 63 as a control group, all aged 20–45 years. Three-day records were collected from participants to estimate the nutrient intake of cases and controls. A body composition analyzer was used to measure body mass index (BMI), body fat (BF), and visceral fat (VF). Biochemical measurements were taken to determine the lipid profile, total testosterone, and serum vitamin D-25-OH. The women’s frequency distribution based on sociodemographic characteristics revealed significant differences within and between the groups. The variations in dietary intake between the PCOS-P and control groups were primarily in terms of total calories, carbohydrates, niacin, and folate, all of which were significantly higher in the PCOS-P group. Dietary fiber, unsaturated fat, vitamin A, vitamin B12, calcium, phosphorus, and selenium, on the other hand, were significantly higher in the control group. A majority of both groups had significantly higher BMI (overweight or obese) and higher BF, but normal VF. According to the findings, testosterone levels in PCOS-Ps were significantly higher than in the control group, but vitamin D-25-OH and high-density-lipoprotein cholesterol (HDL-C) were significantly lower. Age, monthly income, cholesterol, low-density-lipoprotein cholesterol (LDL-C), and testosterone were the fundamental causes impacting women’s anthropometric indices. In conclusion, although both groups were overweight or obese, and differences in calorie and nutrient intake, HDL-C, testosterone, and vitamin D-25-OH levels were observed. The study advises such population groups to limit their consumption of foods high in calories.

## 1. Introduction

Both teenage girls and women who are reproductive suffer from PCOS, a complicated reproductive endocrinology ailment [1]. The underlying cause of PCOS is an ovarian cell defect (most likely theca cells), which results in excessive androgen synthesis as well as clinical and biochemical symptoms [2]. It is a polygenic, multifactorial condition that combines autoimmune disease, a state of inflammation, and dysregulated steroid production with metabolic and psychological issues. In addition to hyperandrogenism, PCOS causes a variety of health problems, such as obesity, insulin resistance (IR), infertility, cardiovascular disease, and many more [3]. Hormonal and reproductive problems include less fertility, problems with follicular maturation, problems with ovulation, and too much luteinizing hormone (LH) production [4]. Although the precise etiology of this illness is still mainly unclear, accumulating evidence points to PCOS as a complex multi-genic condition with significant environmental and epigenetic consequences, including aspects related to diet and lifestyle [5]. Globally, 6–10% of reproductive-aged women are affected by this endocrine–metabolic condition [6].

The variations in PCOS prevalence could be attributed to diverse population studies, their sample size, the age of included women, the location where the subjects were recruited, the criteria used to identify PCOS, and the methodology used to define the criteria [7]. In Saudi Arabia, PCOS differs by area. PCOS was shown to be more common in female students at Taibah University in Madinah. From this cohort of 201 participants, 108 (53.7%) were diagnosed with PCOS, and the majority of them were in the age range of 25–30 years [8]. Further, despite the lack of verified data on the estimated frequency of PCOS in Saudi females, a paper by Bukhari et al. [9] suggested that 31.8% of Saudi females may have the condition. A report [10] also discussed the significance of nutrition and exercise in controlling PCOS symptoms and averting the syndrome’s metabolic consequences. Obesity also worsens the reproductive and metabolic profiles of PCOS [11].

A low socioeconomic condition corresponds to unhealthy behaviors that can prompt obesity, hormonal reactions, or the activation of a genetic predisposition to PCOS symptoms [12]. Due to restricted access to and use of health care, it may also result in a reduction in the condition’s management and treatment [13]. It has been suggested that lifestyle changes can help both the metabolic and reproductive symptoms of PCOS. The sustainable treatment for PCOS appears to be a change in lifestyle to avoid abnormal immune system stimulation and to limit exposure to substances that can cause inflammation [3]. In one study, food intake was measured using a semiquantitative food intake questionnaire [1]. The findings indicated that women with PCOS consume more fat and total energy than women without PCOS. Furthermore, a study has shown that women with PCOS eat a lower proportion of fiber and more foods with a high glycemic index [14]. Studies have found that women with PCOS consume more calories than women without the illness, despite some finding no differences in dietary intake or physical activity [1,15].

Obesity and PCOS have a bidirectional relationship, with overweight women having a higher prevalence of obesity, particularly central or visceral obesity, which exacerbates PCOS’s metabolic and reproductive signs [16]. Although it is not necessary for the diagnosis, obesity is a typical sign of PCOS and is linked to substantial health concerns [17,18]. The most frequent lipid abnormalities in women with polycystic ovarian syndrome include increased levels of triglycerides (TGs), HDL-C, LDL-C, and very low-density-lipoprotein cholesterol (VLDL-C). A study found that vitamin D deficiency was more common among PCOS patients in Saudi Arabia [19]. Kalyanaraman and Pal [20] investigated the pathophysiological importance of vitamin D insufficiency in PCOS, and they discovered that vitamin D deficiency is correlated with poor reproductive outcomes, including PCOS. However, there is little research regarding Saudi women’s food intake and PCOS prevalence. Thus, this study was performed to investigate the connection between food consumption and adipose tissue distribution in Saudi women with POCS and those in good health.

## 2. Materials and Methods

### 2.1. Sample Selection and Size

In 2021, a cross-sectional study with adult female Saudi volunteers (*n* = 105) aged 20–45 years was conducted. Participants were split into two sections: a control group (*n* = 63) and a PCOS patient group (*n* = 42). King Fahad Medical City’s Reproductive Endocrine and Infertility Medicine Department (REIMD) identified the PCOS patients. Females of Saudi nationality who did not have a mental disorder, eating disorder, or chronic disease that affected nutrient metabolism were selected. Women of non-Saudi origin, pregnant or lactating, those with a chronic disease (liver and kidney disease), and those using insulin or any medication that affected appetite or weight and following a special diet, as well as those with a history of malignancy or celiac disease, were excluded. The case–control study’s accuracy and prevalence of satisfaction were considered while calculating the sample size, and the inclusion and exclusion criteria were used to choose the participants conveniently. The study hypothesized that PCOS participants’ dietary intake and body composition differed from those of the control group.

### 2.2. Sociodemographic Data Collection

A panel of nutrition experts scrutinized the method of data collection using a structured questionnaire. Before the study began, the researcher conducted in-person interviews with the participants who had given written, informed consent. The demographic information was gathered via a questionnaire that asked questions about the participant’s age, education, marital status, monthly income, and living standard.

### 2.3. Food Nutrient Analysis

A daily food record method was used to collect the data on food intake. The lead researcher and his assistants gave the participants printed records with instructions, examples, and tables to record the food they had eaten each day for three consecutive days to perform the best dietary consumption analysis possible. Participants documented all dietary intake, including meals, snacks, beverages, sauces, and seasonings, even if it was in small amounts. More food record sheets were distributed to participants who required more space to record what they had consumed. Each record sheet had the researcher’s mobile phone number noted on it, along with instructions about how important it was to be accurate and thorough. This was used if a participant had any inquiries or concerns. Respondents were also instructed to continue with their regular eating routines regardless of the outcome, as the goal was to direct them to the best eating habit based on the results. The responders were given instructions by the researcher and were requested to provide precise descriptions of the food kind, quantity, and preparation method using everyday measurements such as tablespoons, teaspoons, and cups. Three-day records were collected from participants to estimate the nutrient intake of cases and controls (ESHA Food Processor software, version 11.9.13, 2020). The software was utilized to determine nutrient intake once it was updated to include Saudi diets. This software assigned a code to each type of meal, whether it was fresh or cooked. Following an automatic examination of the foods consumed, the program calculated the precise amounts of nutrients for every participant based on their daily consumption over three consecutive days, including calories, carbohydrates, protein, etc. In addition to ingredients, recipes, and other food products, the ESHA Master Database provides nutritional breakdown information for each food item in up to 172 data categories, including water, macronutrients, and micronutrients.

### 2.4. Anthropomorphic Measurement

Anthropometric measurements were performed using a body composition analyzer (AC-CUNIQ BC360, SELVAS Healthcare Inc., Daejeon, Republic of Korea). Electrodes were positioned on the wrists and feet of the standing patients, and measurements were done three times on different days. The BMI and percentages of body and visceral fat were automatically computed when the apparatus was coupled with an ultrasonic height meter. The participants’ palms and soles were cleansed following each measurement, and they fasted for four hours before the test. Enough privacy was provided for the participants. To determine the status of body weight, the BMI (kg/m^2^) was calculated and categorized following WHO classifications [21]. The BF and VF were classified according to the description of Kyle et al. [22].

### 2.5. Blood Sample Collection and Analysis

The nurses took blood samples in the early follicular phase of each patient’s spontaneous or progestin-induced menstrual cycle in the morning after the women had fasted the previous night. The King Fahad Medical City lab received these blood samples. Lipid profile measurements were done with colorimetric kits and a Dimension Xpand Plus Chemistry Analyzer. This profile includes serum TG, total cholesterol, HDL-C, and LDL-C levels. A chemical analyzer device was used to do the testosterone analysis. An automated Cobas e411 analyzer was used to perform the total serum 25-hydroxyvitamin D analysis (Roche Diagnostics, Indianapolis, IN, USA). According to Herrmann et al. [23], a serum level of 25-hydroxyvitamin D below 30 nmol/L signifies vitamin D deficiency, a level between 30 and 50 nmol/L indicates insufficiency, and a level over 50 nmol/L implies sufficiency.

### 2.6. Statistical Analysis

The data were statistically analyzed using SPSS software version 20, and the results are shown as frequencies or as means with standard deviation. The anthropometric data were compared via a chi-squared test. All data for each food item for PCOS patients and the control group were displayed as means ± standard deviations and then compared using a *t*-test with a *p*-value. Linear regression analysis between the sociodemographic characteristics and anthropometric indices was undertaken. Pearson correlation coefficients were used to assess the correlations between anthropometric indices (BMI, BF, and VF), lipid profile, testosterone, and vitamin D-25-OH.

## 3. Results

### 3.1. Socioeconomic Traits

Table 1 depicts the frequency distribution of PCOS-P women and a control group based on sociodemographic characteristics. In both research groups, the age-based frequency distribution of participants showed significant variations. Those aged 29 to 37 years comprised the majority of both the PCOS-P (57.1%) and control groups (46.0%). The bulk of the PCOS-P (50.0%) and control groups (57.1%) had completed a diploma and/or university-level education, while those with only a secondary school education came in second. In terms of monthly income, 47.63% of the PCOS-Ps and 36.51% of the control group earned a monthly income of SAR 6000 to 10,000, which is the highest percentage for both groups. With 92.8% of the PCOS-Ps and 90.50% of the control group, both groups had a significantly higher percentage of married females. In terms of living conditions, more than 90% of both groups lived at their husbands’ house, with only a few living with their parents, the mother, or by themselves.

### 3.2. Nutrient Intake

Table 2 displays the daily nutrient intake data (as an average daily nutrient consumed in a 3-day diary) for PCOS-P women and the control group. The computed average nutrient intake of PCOS-P and the control group were compared via a Student’s *t*-test. The women with PCOS consumed a lot more calories, total carbohydrates, total fat, niacin, and folate on average (2257.15 kcal, 270.89 g, 89.05 g, 24.8 mg, and 235.33 mg, respectively) than the control group (2220.92 kcal, 255.13 g, 85.20 g, 19.45 mg, and 231.25 mg, respectively) (*p* ≤ 0.05). However, the control group consumed significantly (*p* ≤ 0.01) more dietary fiber and phosphorus (31.99 g and 902.80 mg, respectively) than the PCOS-P women (9.75 g and 856.52 mg, respectively). The control group had significantly higher levels of unsaturated fat, vitamin A, vitamin B12, calcium, and selenium (*p* ≤ 0.05).

### 3.3. Anthropometric Indices

The BMI, BF, and VF classification data of the PCOS-Ps and the control group are listed in Table 3. Between the groups, variations in BMI classes were significant (*p* = 0.002). Most of the PCOS-Ps were either overweight (31.1%) or obese (33.3%) with obesity I, whereas most of the control group were either overweight (50.8%) or obese (19.1%) with obesity I as well. Additionally, obesity II and III were observed among the participants, as well as those who were underweight (but these patients were represented at small frequencies). Obese females outnumbered all other classes in the PCOS-Ps, while overweight females outnumbered all the other classes in the control group.

Since the distribution of adipose tissue varied among the participants, respondents’ BMIs, as indices for obesity, may not always accurately reflect the degree of their visceral fat levels. Because of this, BF and VF, in addition to BMI, were employed as indicators to investigate the anthropometric characteristics of the females. According to the body composition analyzer, the BF classes varied among the groups (*p* = 0.035), with the majority of the PCOS-P (42.9%) and control groups (44.4%) having high BF levels, while the minority of the participants in both groups (21.4 and 23.8%, respectively) revealed the highest BF levels. Furthermore, only the control group (4.8%) had a low level of BF. The VF classes were different between the groups (*p* = 0.006). Most of the women in the PCOS group (88.1%) and the control group (87.3%) had normal VF levels, while a few women in those groups had low frequencies of other VF classes.

### 3.4. Lipid Profile, Testosterone, and Serum Vitamin D-25-OH

Table 4 displays the levels of serum lipid profile, testosterone, and vitamin D-25-OH of women in good health and women suffering from PCOS. HDL-C was significantly (*p* ≤ 0.05) higher in the control group (3.58 mmol/L) than in the PCOS-P group (3.58% mmol/L). Furthermore, testosterone levels in the PCOS-P group were significantly (*p* ≤ 0.01) higher (2.12 nmol/L) than those in the control group (1.06 nmol/L). Also, PCOS-P women did not have enough vitamin D-25-OH. Their levels were 39.52 nmol/L, which is significantly (*p* ≤ 0.01) lower than the control group’s levels (56.01 nmol/L). Cholesterol, LDL-C, non-HDL-C, and TG levels varied between the groups, but were not statistically different.

### 3.5. Factors Associated with Anthropometric Characteristics

Table 5 shows the interrelationships between the sociodemographic traits of the examined groups and their anthropometric indices. We used linear regression analysis to find statistical links between the women’s anthropometric measurements (as dependent variables) and their sociodemographic data (as independent variables). We employed the BMI, BF, and VF as markers for the anthropometric characteristics of women. The ages of PCOS-Ps and the control group showed a significant positive correlation (*p* ≤ 0.05 or *p* ≤ 0.01) with anthropometric indices. In both the PCOS-P and control groups, monthly income was positively connected with anthropometric markers and was also statistically significant (*p* ≤ 0.05 or *p* ≤ 0.01). However, the education level was found to have a substantial, positive influence on the BF and VF of the control group (*p* ≤ 0.05). The type of residence and marital status had no discernible effect on anthropometric proxies.

We used the Pearson correlation coefficient to find statistical links between the women’s anthropometric measurements (as dependent variables) and their lipid profile, testosterone, and serum vitamin D-25-OH levels (as independent variables) (Table 6). The findings revealed that cholesterol levels were crucially positively connected with anthropometric indicators in both the PCOS-P and control groups (*p* ≤ 0.05 or *p* ≤ 0.01). Triglyceride levels were significantly and positively linked to PCOS-P BF and VF, as well as to the BMI and BF in the control group (*p* ≤ 0.05) (Table 6). The LDL-C level was correlated significantly (*p* ≤ 0.05 or *p* ≤ 0.01) with the PCOS-P anthropometric indicators, but it was correlated significantly only with the BMI and BF in the control group. Only the anthropometric proxies of the PCOS-Ps were significant (*p* ≤ 0.05 or *p* ≤ 0.01) and favorably associated with testosterone levels (Table 6).

## 4. Discussion

This research addressed the differences in food intake and anthropometric variables between women with PCOS-P and those in good health through a cross-sectional case–control study. Based on sociodemographic factors, the study’s results showed that there were significant differences within the group for all features in the frequency distribution of the females. This was shown by the chi-squared test. The results also demonstrated that most members of both groups were married, between the ages of 29 and 37 years, had a diploma or university degree (or a respectable monthly income), and resided in their husband’s house. Variations in living environments and tribes might be responsible for variations in sociodemographic traits. Despite the participants’ high socioeconomic status, their health knowledge appears to be poor. This is unexpected, because research indicates that low socioeconomic status is linked to unhealthy habits that can cause obesity, set off hormonal reactions, or activate a genetic susceptibility to PCOS symptoms [12]. It can also result in a lack of access to and use of health care, which can impede PCOS management and treatment [13].

Reproductive-aged females are the main victims of PCOS, a complicated endocrine ailment [15,24]. The patients in our study were mostly between the ages of 20 and 45, indicating that they were in their prime reproductive years. This age group has a 4–18% prevalence of PCOS, which is linked to hormonal, metabolic, and reproductive disruptions, as well as an increased risk of pregnancy issues [25,26,27]. Similar demographic characteristics were found in a study on the psychological toll of PCOS for Saudi women, with the majority of patients being well-educated, married, and falling into the 28–35 age range [28].

The main differences in dietary intake between the PCOS-P and control groups were in total calories, carbohydrates, niacin, and folate, all significantly higher in the PCOS-Ps. Conversely, dietary fiber, unsaturated fat, vitamins A and B12, calcium, phosphorus, and selenium were found at significantly higher levels in the control group. A semiquantitative food intake questionnaire was used in a study to assess food consumption [1], and the findings revealed that women with PCOS consume more fat and total energy than women without PCOS. Furthermore, some studies have found that PCOS women consume more foods with a high glycemic index and less fiber [14]. While some studies found no differences in dietary intake or physical activity, others discovered that women with PCOS eat more calories than those without the illness [1,15].

Cowan et al. [29] came to a similar conclusion: PCOS patients consume more fat and calories than non-PCOS patients, irrespective of their dietary patterns. This finding may be connected to the disorder. Furthermore, some previous studies have found that women with PCOS consume more calories than women without the illness, while other studies have not found any significant variations in dietary consumption or physical activity levels between PCOS-Ps and control groups [1,30,31]. On the other hand, we observed that PCOS-Ps consumed significantly less dietary fiber. This agreed with the findings of previous research [32] stating that PCOS patients and non-PCOS patients consumed significantly less than the recommended daily fiber. The greatest risk of mineral deficit came from calcium, phosphorus, and magnesium shortages in PCOS-afflicted women, whereas tested women were at risk of vitamin deficiency due to insufficient folic acid, vitamin C, vitamin D, and vitamin B12 [33]. According to Turner-Mcgrievy et al. [34], overweight women with PCOS-linked infertility consume inadequate amounts of whole grains, fiber, and iron in their diets. These eating habits are incompatible with maintaining a healthy body weight and having low PCOS-associated quality of life scores. In general, women with higher androgen levels, regardless of PCOS, have stronger appetites for foods rich in fats and carbohydrates [35], and they may consume more of these foods [14]. This could also be a factor in the higher prevalence of obesity among PCOS patients when compared to non-PCOS patients.

Since there are individual differences in the distribution of adipose tissue (BF), the BMI as a measure of obesity may not always accurately reflect a respondent’s VF level. This is because VF, which is a type of hidden fat that is wrapped around internal organs like the liver and intestines, is stored deep within the abdomen. For this reason, measurements of BF and VF were made in addition to BMI. According to the findings of this study, the majority of both groups had very high BMIs (indicating that they were overweight or showed obesity) and high BF but normal VF. The prevalence of reported PCOS increased significantly with increasing BMI. Every unit rise in BMI has resulted in a significant increase in the prevalence of reported PCOS. The average BMI during the follow-ups was shown to have the highest link with self-reported PCOS status. The average BMI during the follow-ups was shown to have a paramount link with the self-reported PCOS condition. However, only a few cross-sectional studies in specific populations have examined the relationship between BMI and PCOS prevalence, and those conducted have produced contradictory results [36,37]. Overweight PCOS patients experience more severe PCOS symptoms than normal-weight PCOS patients because android obesity, also known as abdominal obesity, is a prevalent trait in PCOS-Ps, which might be associated with PCOS problems [29]. According to Asdaq et al. [38], PCOS is more frequent in Saudi Arabia, in line with its worldwide frequency, and PCOS-afflicted females have a different body composition than normal females, favoring higher waist-to-hip ratio (WHR), BMI, body weight, and body fat. Conversely, PCOS-afflicted females had lower bone mineral density than their non-PCOS counterparts [38]. Compared to women without PCOS, PCOS-afflicted women seem to acquire weight more frequently and are more likely to be overweight or obese [10,39].

In this paper, HDL-C levels in PCOS-Ps were significantly lower than in the control group, although LDL-C levels were statistically similar. Coinciding with our findings, Jahan et al. [40] found that in comparison to a control group, HDL-C levels in PCOS-afflicted women were lower, but total LDL-C values were higher. Previous research has not discovered significant variations in TG, LDL-C, HDL-C, or total cholesterol between PCOS-Ps and control groups [41]. Certain researchers have discovered that obesity can account for alterations in LDL-C and HDL-C levels in PCOS-Ps and that the only apparent secondary effect of PCOS-related insulin resistance is a little elevation in total triglyceride levels [42,43]. Sánchez-Ferrer et al. [44] discovered that LDL-C and total cholesterol levels were statistically the same in the PCOS-P and control groups. In comparison to the control group, PCOS-afflicted females had higher TG levels and lower HDL-C levels. Furthermore, in a lipid profile study, Luo et al. [45] reported that the most common abnormalities were low HDL-C and high TG. When compared to normal women, the PCOS group had significantly lower HDL-C levels, but no significant variations in total cholesterol, LDL-C, or TG levels were observed. In addition, Chien et al. [18] found that PCOS patients revealed a rise in levels of LDL-C and TG and a decline in levels of HDL-C. PCOS women (estimated to be >70%) have lipid abnormalities, such as low HDL-C and cholesterol, as well as high TG and LDL-C levels [46].

Compared to women without PCOS, there was a significant increase in testosterone levels and a decrease in vitamin D-25-OH levels. Regarding testosterone levels, the results of this investigation confirmed those of other earlier research that discovered increased testosterone levels in those with PCOS in contrast to the control group [44,47]. Moreover, compared to women with healthy ovaries, women with PCOS have lower LDL-C levels and greater levels of free testosterone, triglycerides, and C-peptides, suggesting that they also exhibit PCOS’s metabolic and endocrine disorders [48]. Our results endorse earlier research that comparing PCOS patients to a control group revealed decreased and insufficient serum levels of vitamin D-25-OH. According to several studies, both PCOS and non-PCOS women frequently have vitamin D insufficiency [19,49,50].

Additionally, vitamin D deficiency was reported to be highly prevalent in Saudi PCOS patients, according to a study [51]. Kalyanaraman and Pal [20] investigated the pathophysiological significance of low vitamin D levels in PCOS and discovered that vitamin D is linked to scanty fertility results, which include PCOS. Recent research suggests that vitamin D administration may benefit metabolic, endocrine, and fertility aspects in PCOS patients [52,53]. However, Daghestani et al. [54] did not discover any inadequate vitamin D supply or variation in vitamin D levels between the two groups under investigation. Other studies have found no differences between PCOS cases and controls [31,55]. Variations in a patient’s age, BMI, skin tone, nutrition style, and physical activity, as well as variations in study design, such as sample size and methodological choice, are the main causes of such discrepancies in research findings.

The participants’ anthropometric traits, like age and monthly income, were favored by the independent factors, as evidenced by the substantial positive correlation found between the anthropometric indices as dependent variables and sociodemographic traits as independent variables. Education level, on the other hand, was found to favor the control group’s body proxies. In agreement with this finding, Tayyem et al. [56] discovered that age had a positive connection with “healthy” dietary patterns and that educated women are more inclined to commit to a healthier eating pattern. In their investigation of the tie between age and BMI, Jeong et al. [57] found that although the average BMI increase was higher in younger individuals, BMI increased with age until the seventh and eighth decades of life, so they concluded that BMI increases in young adults but decreases with age. In line with our findings regarding the relationship between income and anthropometric indices, Hunter et al. [58] reported that in females, the upsurge in the consumption of fast foods and energy-dense meals are the major contributors to the global obesity epidemic, disproportionately affecting the poor. According to their report, people with limited financial resources can choose a diet that offers the most calories for the least money, making healthy diets unaffordable for the poor. Furthermore, Lawson et al. [59] found that high income impacts female BMI and other anthropometric traits, particularly in rich countries.

Although both groups of women had diplomas and university degrees, the education level favored the BF and VF of the control group, but not the PCOS-Ps. A study found a positive relationship between education and obesity; furthermore, it has also shown that the burden of obesity is shifting towards the less educated, which supports this finding [60]. Moreover, Jimenez-Mora et al. [61] discovered that women’s overall and abdominal obesity were negatively correlated with education. Furthermore, compared to their counterparts, women with higher levels of education are more likely than their counterparts to practice preventive health behaviors like frequent exercise and eating a healthy diet. They may also be less inclined to have high uniformity, which is linked to being overweight or obese [62].

However, some investigated variables (women’s anthropometric traits) were strongly linked to biochemical variables (triglycerides, testosterone, and vitamin D-25-OH levels). This meant that testosterone, triglycerides, LDL-C, and cholesterol supported the women’s anthropometric traits. This study found a favorable correlation between the LDL-C, BMI, BF, VF, and cholesterol levels of both groups. Nevertheless, the vitamin D, BMI, BF, and VF levels of the groups did not correlate, although testosterone was favorably correlated with these variables in the PCOS-Ps. Kiranmayee et al. [46] found that there were strong negative links between BMI and HDL-C in PCOS women who were either normal weight, overweight, or obese. There were also strong positive links between triglycerides and BMI and between triglycerides and waist circumference across the whole study group. In a study by Blagojevic et al. [63], PCOS-afflicted females had lower HDL-C levels and greater BMI. Further, Boshku and Panova [64] noticed favorable associations between LDL cholesterol and BMI, WC, HC, WHTR, and TC. Earlier studies disclosed that BMI substantially impacted LDL, HDL, and non-HDL cholesterol levels in women with PCOS [18,45].

According to Mongraw-Chaffin et al. [65], testosterone bioavailability is substantially related to VF and is unaffected by age, total body obesity, or cardiovascular risk factors. BMI is not a particularly accurate indicator of adiposity level; hence, BF and VF were included in this study. Many researchers have looked into the relationship between adiposity (determined by BMI) and amounts of vitamin D in PCOS-afflicted women. Vitamin D was not linked to the PCOS-P or control groups’ BMI, BF, or VF in this study. In contrast, a different study found a negative correlation between vitamin D levels and BMI [49]. Moreover, an investigation reported an inverse link between vitamin D amounts and BMI [66]. In contrast, a study on PCOS-afflicted females denied the existence of a correlation between levels of vitamin D and BMI or WHR [67]. The large range in vitamin D levels due to the non-representative sample size, the use of BMI to define obesity, the small number of women with PCOS who were vitamin D-deficient, and the different ways that vitamin D levels were measured could all explain the different results. Additionally, no correlation between vitamin D insufficiency and PCOS in young females was discovered in a study that assessed BMI [68]. Based on these results and the reviewed literature, BF may have a direct impact on the decrease in vitamin D in PCOS. Also, the study realized an upward association between BF and VF and the TG level, a prominent metabolic risk factor in the pathogenesis of PCOS and linked to an elevated risk of cardiovascular disease.

## 5. Conclusions

The data of this study illustrated that the participants (both with PCOS and the control group) differed regarding sociodemographic factors. Compared to their counterparts, PCOS-Ps consumed more calories and calorie-rich nutrients while consuming less calcium and phosphorus. Both the PCOS-P and control groups were overweight or obese, with high body fat and normal visceral fat. PCOS women had lower HDL-C and vitamin D-25-OH mean values, but higher testosterone levels than the control group. For both groups, age and monthly income were identified as factors associated with increased anthropometric characteristics, as well as cholesterol and LDL-C. However, testosterone was identified as a factor associated with the improved anthropometric characteristics of PCOS women and not the control group. The researchers discovered that BF and VF were positively related to TG levels. TG is a metabolic risk factor that is frequently present in the pathogenesis of PCOS and is associated with an increased probability of exposure to cardiovascular disease. As a result, patients with PCOS will face challenges with lifestyle changes and will need to address these issues with caution. To cover all nutrient requirements, a highly varied diet is advised; nevertheless, to prevent weight gain, the recommendations to diversify the diet should be implemented by limiting total calorie intake. Determining the relative contributions of weight, metabolism, and lifestyle factors to PCOS needs more research as well. It is also advised to raise awareness of PCOS among women and encourage them to communicate with health-care providers to initiate treatment.

## 6. Study Limitations

Because this study targeted a particular area of Saudi Arabia, its relatively small sample was a drawback. Additionally, given the country’s vastness, it would have been difficult to conduct the study nationwide. As part of the cross-sectional study, it is important to use caution when interpreting the results. In addition, since the bulk of participants did not exercise, it was difficult to include physical activity. The sample was ultimately small due to the limited number of cases available. As the study coincided with the beginning of the end of the ban that accompanied the COVID-19 pandemic, it was challenging to recruit an equal number of PCOS women and controls. Due to dietary and physiological differences between nations, non-Saudis were also left out.

## Figures and Tables

**Table 1:** Frequency distribution of the participants according to sociodemographic data.

Sociodemographic Characteristics	PCOS-Ps		*p*-Value	Control Group		*p*-Value
	Frequency	Percent		Frequency	Percent	
Age (years)						
20–28	11	26.2	0.044	13	20.7	0.001
29–37	24	57.1		29	46.0	
More than 37	7	16.7		21	33.3	
Education						
Illiterate	2	4.80	0.001	2	3.20	0.003
Secondary school	18	42.80		24	38.10	
Diploma and university	21	50.00		36	57.10	
Graduate studies	1	2.40		1	1.60	
Monthly Income						
1000–3000 SR	5	11.90	0.036	6	9.52	0.041
3000–6000 SR	14	33.33		22	34.92	
6000–10,000 SR	20	47.63		23	36.51	
>10,000 SR	3	7.14		12	19.05	
Marital status						
Single	1	2.40	0.005	6	9.50	0.0001
Married	39	92.80		57	90.50	
Divorced	2	4.80		-	-	
Living condition						
Live with both parents	1	2.38	0.046	5	7.93	0.019
Live with mother	1	2.38		1	1.59	
Live in husband’s home	39	92.86		57	90.48	
Live alone	1	2.38		-	-	

PCOS-Ps = polycystic ovary syndrome patients.

**Table 2:** Average daily intake of nutrients (daily records for 3 different days) for PCOS-Ps and the control group.

Nutrient Intake	PCOS-Ps	Control Group	Difference	*t*-Test	*p*-Value
	X ± SD	X ± SD			
Calories (kcal)	2257.15 ± 295.95	2220.92 ± 272.18	36.23	0.48	0.026 *
Total protein (g)	109.28 ± 41.25	107.18 ± 40.79	2.1	0.54	0.591
Total carbohydrates (g)	270.89 ± 72.66	255.13 ± 72.86	15.76	1.58	0.031 *
Dietary fiber (g)	9.75 ± 6.17	31.99 ± 19.65	−22.24	1.40	0.006 **
Total fat (g)	89.05 ± 34.75	85.20 ± 28.22	3.85	0.62	0.044 *
Saturated fat (g)	26.44 ± 12.68	25.17 ± 13.02	1.27	0.28	0.081
Unsaturated fat (g)	29.61 ± 19.56	33.75 ± 23.64	−4.14	1.95	0.050 *
Cholesterol (mg)	390.49 ± 229.09	361.53 ± 212.81	28.96	0.42	0.569
Vitamin A µg (RE)	396.99 ± 163.91	478.74 ± 184.77	−81.75	1.29	0.022 *
Vitamin B1 (mg)	1.25 ± 0.71	1.16 ± 0.61	0.09	0.79	0.433
Vitamin B2 (mg)	1.82 ± 1.47	1.68 ± 1.32	0.14	0.13	0.901
Niacin (mg)	24.80 ± 7.02	19.45 ± 15.45	5.35	1.09	0.080 *
Vitamin B6 (mg)	1.37 ± 1.27	1.12 ± 0.68	0.25	1.08	0.285
Vitamin B.12 (mg)	3.52 ± 2.55	6.17 ± 2.67	−2.65	0.99	0.027 *
Vitamin E (mg)	3.44 ± 3.47	4.11 ± 1.74	−0.67	0.86	0.393
Vitamin D (mcg)	5.84 ± 1.23	6.08 ± 1.49	0.24	0.74	0.293
Folate (mg)	235.33 ± 161.49	231.25 ± 16.83	4.08	0.28	0.047 *
Calcium (mg)	690.59 ± 326.15	820.48 ± 507.18	−129.89	1.96	0.050 *
Copper (mg)	1.10 ± 2.24	1.27 ± 3.76	−0.17	0.48	0.636
Iron (mg)	13.22 ± 5.90	12.19 ± 8.07	1.03	0.97	0.123
Phosphorus (mg)	856.52 ± 365.72	902.80 ± 76.21	−46.28	0.81	0.004 **
Selenium (mg)	71.85 ± 56.82	75.92 ± 45.44	−4.07	2.63	0.029 *
Zinc (mg)	8.49 ± 6.24	7.38 ± 5.77	1.11	3.32	0.841

PCOS-P = polycystic ovary syndrome patients; ** *p* ≤ 0.01; * *p* ≤ 0.05 (Student’s *t*-test).

**Table 3:** The body mass index (BMI), body fat (BF), and visceral fat (VF) of the PCOS patients and the control group.

Anthropometric Index	PCOS-Ps		Control Group	
	Frequency	Percentage	Frequency	Percentage
BMI (kg/m^2^)				
Underweight (<18.5)	1	2.4	1	1.6
Normal (18.5–24.9)	10	23.8	10	15.9
Overweight (25–29.9)	13	31.0	32	50.8
Obesity I (30–34.9)	14	33.3	12	19.1
Obesity II (35–39.9)	3	7.1	4	6.3
Obesity III (≥40)	1	2.4	4	6.3
BF (%)				
Low (<11%)	–	–	3	4.8
Normal (11–21.9%)	15	35.7	17	27.0
High (22–27%)	18	42.9	28	44.4
Very high (>27%)	9	21.4	15	23.8
VF (%)				
Low (<10)	3	7.1	3	4.8
Normal (10–14.9)	37	88.1	55	87.3
High (15–20)	1	2.4	4	6.3
Very high (>20)	1	2.4	1	1.6

PCOS-Ps: polycystic ovary syndrome patients; chi-squared of BMI (*p* = 0.002), BF (*p* = 0.034), and VF (*p* = 0.006).

**Table 4:** The average total levels of serum cholesterol, TG, LDL-C, HDL-C, non-HDL-C, testosterone, and vitamin D-25-OH of PCOS-Ps and the control group.

Variables	PCOS-Ps	Control Group	*t*-Test	*p* Value
	X ± SD	X ± SD		
Cholesterol (mmol/L)	4.93 ± 0.79	4.67 ± 0.74	0.87	0.390
Triglyceride (mmol/L)	1.45 ± 1.03	1.82 ± 2.19	0.31	0.756
HDL-C (mmol/L)	1.30 ± 0.30	3.58 ± 18.12	1.03 *	0.032
LDL-C (mmol/L)	3.23 ± 0.76	3.16 ± 0.77	0.73	0.470
Non-HDL-C (mmol/L)	3.62 ± 0.86	3.52 ± 0.75	0.16	0.875
Testosterone (n mmol/L)	2.12 ± 0.98	1.06 ± 0.33	6.48 **	0.002
Vitamin D-25-OH (n mmol/L)	39.52 ± 34.29	56.01 ± 39.63	2.05 **	0.007

PCOS-P = polycystic ovary syndrome patients; ** *p* ≤ 0.01; * *p* ≤ 0.05 (Student’s *t*-test).

**Table 5:** Linear regression analysis between the sociodemographic characteristics and anthropometric indices of PCOS-Ps and the control group.

Dependent Variable/ Independent Variable	PCOS-Ps						Control Group					
	BMI		BF		VF		BMI		BF		VF	
	(β, r^2^)	*p*-Value	(β, r^2^)	*p*-Value	(β, r^2^)	*p*-Value	(β, r^2^)	*p*-Value	(β, r^2^)	*p*-Value	(β, r^2^)	*p*-Value
Age	(0.142 **, 0.039)	0.003	(0.330 *, 0.049)	0.050	(0.218 **, 0.132)	0.001	(0.353 *, 0.006)	0.049	(0.222 *, 0.011)	0.047	(0.282 *, 0.029)	0.027
Education	(0.191, 0.038)	0.066	(0.109, 0.005)	0.770	(0.058, 0.007)	0.304	(0.110, 0.056)	0.238	(0.367 **, 0.054)	0.005	(0.267 *, 0.045)	0.024
Monthly income	(0.162 *, 0.007)	0.026	(0.166 *, 0.034)	0.047	(0.170 *, 0.023)	0.017	(0.205 *, 0.010)	0.050	(0.118 *, 0.213)	0.039	(0.225 **, 0.023)	0.006
Marital status	(0.122, 0.003)	0.758	(0.038, 0.001)	0.147	(0.008, 0.0001)	0.996	(0.154, 0.003)	0.347	(0.281, 0.037)	0.070	(0.072, 0.001)	0.334
Living condition	(0.285, 0.025)	0.531	(0.206, 0.043)	0.424	(0.591, 0.047)	0.323	(0.021, 0.004)	0.306	(0.414 *, 0.0380	0.026	(0.372, 0.002)	0.276

PCOS-P = polycystic ovary syndrome patients; * *p* ≤ 0.05; ** *p* ≤ 0.01. Regression coefficients (β), partial r^2^ for independent variables of interest.

**Table 6:** Pearson correlation coefficients between the lipid profile, testosterone, vitamin D-25-OH serum, and anthropometric markers of PCOS-Ps and the control group.

Dependent Variable/ Independent Variable	PCOS-Ps						Control Group					
	BMI		BF		VF		BMI		BF		VF	
	r	*p*-Value	r	*p*-Value	r	*p*-Value	r	*p*-Value	r	*p*-Value	r	*p*-Value
Cholesterol	0.346 **	0.003	0.583 *	0.023	0.238 **	0.001	0.240 *	0.025	0.088 *	0.044	0.123 *	0.031
Triglyceride	0.341	0.431	0.104 *	0.017	0.205 *	0.039	0.182 *	0.048	0.121 *	0.035	0.142	0.524
HDL-C	−0.150	0.342	−0.172	0.275	−0.105	0.508	−0.053	0.680	−0.005	0.969	−0.104	0.419
LDL-C	0.533 **	0.009	0.109 **	0.005	0.269 *	0.050	0.113 **	0.002	0.047 *	0.001	0.101	0.004
Non-HDL-C	0.062	0.695	0.128	0.421	0.070	0.659	0.053	0.680	0.005	0.969	0.104	0.419
Testosterone	0.152 *	0.027	0.128 **	0.001	0.217 *	0.049	0.046	0.719	0.069	0.589	0.037	0.773
Vitamin D-25-OH	−0.179	0.208	−0.126	0.079	−0.195	0.056	−0.130	0.089	−0.113	0.449	−0.121	0.078

PCOS-Ps = polycystic ovary syndrome patients; * *p* ≤ 0.05; ** *p* ≤ 0.01.

## Data Availability

The datasets used and analyzed during the current study are available from the corresponding authors upon reasonable request.
